# Viewers can keep up with fast subtitles: Evidence from eye movements

**DOI:** 10.1371/journal.pone.0199331

**Published:** 2018-06-19

**Authors:** Agnieszka Szarkowska, Olivia Gerber-Morón

**Affiliations:** 1 Centre for Translation Studies, University College London, London, United Kingdom; 2 Institute of Applied Linguistics, University of Warsaw, Warsaw, Poland; 3 Department of Translation and Interpreting & East Asian Studies, Faculty of Translation and Interpreting, Universitat Autònoma de Barcelona, Barcelona, Spain; Aston University, UNITED KINGDOM

## Abstract

People watch subtitled audiovisual materials more than ever before. With the proliferation of subtitled content, we are also witnessing an increase in subtitle speeds. However, there is an ongoing controversy about what optimum subtitle speeds should be. This study looks into whether viewers can keep up with increasingly fast subtitles and whether the way people cope with subtitled content depends on their familiarity with subtitling and on their knowledge of the language of the film soundtrack. We tested 74 English, Polish and Spanish viewers watching films subtitled at different speeds (12, 16 and 20 characters per second). The films were either in Hungarian, a language unknown to the participants (Experiment 1), or in English (Experiment 2). We measured viewers’ comprehension, self-reported cognitive load, scene and subtitle recognition, preferences and enjoyment. By analyzing people’s eye gaze, we were able to discover that most viewers could read the subtitles as well as follow the images, coping well even with fast subtitle speeds. Slow subtitles triggered more re-reading, particularly in English clips, causing more frustration and less enjoyment. Faster subtitles with unreduced text were preferred in the case of English videos, and slower subtitles with text edited down in Hungarian videos. The results provide empirical grounds for revisiting current subtitling practices to enable more efficient processing of subtitled videos for viewers.

## Introduction

Together with an ever-growing demand for subtitles in digital media, we are witnessing an increase in subtitle speeds. Yet, despite the ubiquity of subtitling, little is known about optimum subtitle speeds and their impact on cognitive processing. The pervasiveness of subtitling, coupled with a significant shift from slow to increasingly high subtitle speeds [1, 2], offer a timely opportunity to examine the effects of speed on viewers’ processing of subtitled videos.

Two main types of subtitling can be distinguished: intra- and interlingual [3]. Intralingual subtitling (also known as captioning) contains a written version of dialogues in the same language (e.g., English to English), whereas interlingual subtitling is a translation of a foreign dialogue (e.g., English to Dutch). Upon careful review of the subtitling literature, it becomes apparent that in interlingual subtitling, it is part and parcel of the process to condense and reduce the text–the underlying assumption is that because viewers can integrate information from subtitles with that coming from images and soundtrack, verbatim translation is not necessary [3]. Another reason for text reduction stems from reading speed requirements, which are meant to allow viewers to comfortably follow both the subtitles and on-screen action [3, 4]. In contrast, text reduction is not welcome in intralingual subtitling, mainly produced for the deaf and hard of hearing (SDH), who demand to have a verbatim and “uncensored” version of the dialogue to be on a par with hearing people, as well as to avoid mismatches between subtitles and speakers’ lip movements [5–8].

### Subtitle speed

Subtitle speed, also referred to as ‘reading speed’ [2] or ‘presentation rate’ [9], is usually measured in either characters per second (cps) or words per minute (wpm). Given the differences in the length of words in different languages, in the audiovisual translation industry the cps measure is used more often, as it is considered more accurate across languages [10, 11]. Studies on English-to-English SDH, however, have traditionally used the wpm measure [12].

The most widely known rule on the speed of interlingual subtitles–“the six-seconds rule”–stipulates that a full two-line subtitle should be displayed for six seconds in order for an average viewer to be able to read it [3, 13]. The six-seconds rule is equivalent to approximately 140–150 wpm or 12 cps [3, 10]. While the origins of the rule are difficult to trace–d'Ydewalle, Muylle [14] even state that “nobody seems to know how the six-second rule was arrived at”–it is the golden standard recommended in subtitling textbooks [2, 3].

The prevalence of the six-seconds rule may be rooted in the belief that fast subtitle speeds will not allow viewers to follow both the subtitles and the on-screen action [3]. However, how much time do viewers actually spend reading subtitles and watching the images? This can be assessed using the concepts of absolute reading time and proportional reading time [15]. *Absolute reading time* is measured in seconds and it is the actual time spent on reading the subtitle. For instance, a viewer can spend 4 seconds reading a subtitle displayed for 6 seconds, which leaves them 2 seconds to follow the on-screen action in the film. *Proportional reading time* is measured in percentages and is the proportion of the total subtitle display time during which the viewer is actually gazing at the subtitle. Thus, if a reader looks at the 6-second-subtitle for 4 seconds, their proportional reading time is 66%. Longer subtitle display times have been found to increase the absolute reading time but decrease the proportional reading time [15, 16]. On the one hand, this finding may suggest that longer subtitle display times can benefit viewers by giving them more time to follow the on-screen action. On the other hand, however, it is plausible that when faced with fast subtitles, viewers simply read them more efficiently and, ultimately, do not need longer display times.

Subtitle speeds are not set in stone; they differ from country to country and even from company to company [2]. In interlingual subtitling, various countries have followed different traditions on subtitle speeds, ranging from 12 cps on television in Scandinavian countries [4], 15–16 cps in Central Europe [1], to 17–20 cps in global online streaming services [17]. In English intralingual SDH, the typical recommended speed is up to 180 wpm [18, 19], which for the English language is equivalent to 15 cps (assuming that an average word has 5 characters).

From a historical perspective, subtitle speeds have been on the rise in both intra- and interlingual subtitling [2, 3]. In English SDH, subtitles evolved from being a highly reduced and simplified version of the dialogue in the 1970s and 1980s, aimed primarily at prelingual deaf viewers [20], to the current forms of verbatim uncondensed text–a direct result of viewer demands, technological developments and subtitle production costs [21]. In interlingual subtitling, modern viewers are presented with subtitles that are longer than before (a rise from 32 to 42 characters per line) and faster (from 12 to 17–20 cps). In spite of these dramatic changes in subtitle speeds, relatively little attention has been given to their impact on viewers.

Subtitle speeds have been studied extensively in the context of English-to-English SDH [7, 12, 22–31]. As early deafness can be a predictor of poor reading [32, 33], it has been advocated that speed in SDH be slow and that text be edited down [5, 34, 35]. Yet, other studies demonstrated no benefit of slowing down subtitles [6, 9, 27, 36, 37], showing that edited subtitles contain reduced text and fewer cohesive links and, as such, may be difficult to process [38].

In contrast to the vast body of research on subtitle speed in SDH, research on speed in interlingual subtitling has been limited [4] and has possibly become obsolete now. The earliest studies on speed in subtitling with hearing viewers go back to the early 1980s in Belgium. To the best of our knowledge, the first study was undertaken by Muylaert and colleagues [39], using a quite imprecise measurement of subtitle speed: “longer than usual”, “shorter than usual” and “normal speed”. In addition to speed, the authors tested other parameters, including unusual line breaks and difficult vocabulary, conflating the parameters and thus rendering the results problematic. They found that the proportional reading time of “normal” subtitles was 55%, slower subtitles 57% and faster subtitles 68%. Furthermore, another study [14] tested the four-, six- and eight-seconds rules among Dutch viewers and found that the proportional reading time decreased linearly with longer display times: fast, four-second subtitles were looked at for 28% of subtitle display time, six-second subtitles for 23% and slow, eight second subtitles for 21%. Two years later, in what is probably the most often cited study in support of the six-seconds rule, d'Ydewalle and colleagues [16], using the same materials as [14], reported that the shorter the display time of subtitles, the more complaints subjects had about the speed. The subjects also experienced one-line subtitles as faster than two-line subtitles. The authors then tested the correlation between the number of lines (1 vs. 2) and the three speeds (4-, 6-, and 8-seconds) and found that “the only combination where subjects report an appropriate timing is two-line/six-seconds rule” [14]. This statement has often been taken as the empirical validation to support the six-seconds rule that pervades the subtitling industry until now.

### The role of film soundtrack

The reading process of subtitles is dependent on whether viewers can understand the language of the film soundtrack. If they do, it is plausible to assume they would spend less time reading the subtitles, as they would largely draw on the auditory information in their processing of the film dialogue. However, should viewers be unfamiliar with the language of the film soundtrack, we may expect that they would rely more on the subtitles and their subtitle reading time would be longer.

Investigations into the role of the language of the film soundtrack and the language of the subtitles on the processing of subtitled videos and viewers’ visual attention have produced conflicting results [40–46]. On the one hand, some studies [16] found that time spent gazing at subtitles does not change when the soundtrack is muted or when viewers know the language of the soundtrack. Others, on the other hand, reported that less time was spent in the subtitles when the audio was present compared to the no audio condition [43], and found a more regular subtitle reading pattern in videos with interlingual native language subtitles where the soundtrack was in a foreign language unknown to the viewers than in the case of foreign subtitles with the soundtrack in viewers’ native language [47]. Viewers watching intralingual subtitles in their mother tongue skipped them more often than interlingual subtitles with the soundtrack in a foreign language [9]. Finally, watching a film in an unknown language was reported to involve more cognitive effort than watching a film in a familiar language but found no differences in the time spent in the subtitle area as a function of the presence of the soundtrack or the proficiency in the language of the soundtrack [48].

Given that European TV channels and cinemas are largely dominated by English-language productions [49], a large volume of subtitles on the audiovisual translation market are translations from the English language [50]. English is also the best known and the most studied foreign language in the EU [51]. Taken together, this means that many viewers are not only able to understand what is being said in the film without any translation but can also compare the original English dialogue with subtitles. Yet, although in theory such viewers do not need subtitles to follow the dialogue, it has been found that they read subtitles anyway [47, 52]. This phenomenon has been attributed to the dominance of the visual modality [52], the dynamic nature of moving subtitles as they quickly appear and disappear on screen [47, 53] and to the attractiveness and saliency of text even if it is presented in an unfamiliar language [54]. Considering the ubiquity of subtitled content in the world today, it is important to determine the exact role of the language of the film soundtrack on the processing of subtitled videos.

### Experience with subtitling

Previous experience with subtitling–as opposed to other types of audiovisual translation such as dubbing or voice-over–may also affect the way people process subtitled videos. Traditionally, some countries such as Spain, Germany or France have used dubbing to translate foreign films, whereas in others, such as Belgium or Sweden, the preferred mode has been subtitling [55]. However, even in traditionally dubbing countries like Spain, the preference for subtitling against dubbing is now increasing, together with proficiency in foreign languages [56].

Given that, unlike dubbing, subtitling involves dividing viewers’ attention between following the action in the center of the screen and reading the subtitles at the bottom, it may seem that subtitling would be more cognitively taxing for viewers, especially those who are not accustomed to it. However, research has demonstrated that dubbing and subtitling are both effective and enjoyable [55, 56], that there is no trade-off between processing images and subtitles [57] and that the reading of subtitles is automatic [52]. The question remains, however, whether people from what are traditionally considered to be dubbing countries have different strategies to cope with subtitles compared to people more accustomed to subtitling.

### Overview of the current study

The primary goal of the current study is to investigate whether people can keep up with fast subtitles. Therefore, we presented participants with videos subtitled at different speeds and assessed the effects of speed on comprehension, cognitive load, enjoyment, scene and subtitle recognition, reading patterns, preferences as well as reading experience. We were also interested in whether the processing of videos is related to viewers’ knowledge of the language of the film soundtrack as well as their previous experience with subtitling. With these goals in mind, we conducted two experiments with three groups of subjects from different linguistic backgrounds: Spanish, who come from what is traditionally considered a dubbing country; Polish, who are familiar with subtitling; and English, with not much experience in interlingual subtitling. In Experiment 1 we showed people films with the soundtrack in Hungarian to encourage them to read the subtitles. We chose Hungarian as a language dissimilar to any of the native languages of the study participants, who were pre-screened not to know any Hungarian. We used videos dubbed into Hungarian, but originally made in English in order to be able to control the content of subtitle translation into Polish, Spanish and English. Experiment 2 compares the same dependent variables and participants but uses films in English, a language that participants could understand.

We hypothesized that with higher subtitle speeds (20 cps), people may experience increased cognitive load, resulting in more effortful viewing experience, lower comprehension, higher frustration and less enjoyment. To measure cognitive load, we used two types of data: self-reports and eye tracking. Self-reported cognitive load was dissected into three indicators: difficulty, effort and frustration [42]. We expected that fast subtitles would be deemed more cognitively demanding, i.e. more difficult, effortful and frustrating, than slow and medium-paced subtitles. In terms of eye tracking, if subtitles are (too) fast, viewers may spend an excessive amount of time in the subtitle area at the cost of on-screen action, which should be evidenced by longer mean fixation duration, more fixations and longer time spent in the subtitle. On the other hand, extensive text editing, which is inextricably linked with the process of creating slow subtitles, may lead to missing cohesive links and high text condensation, and in consequence may result in additional cognitive effort “to make assumptions to fill in what is missing” [38]. This could, paradoxically, contribute to higher cognitive effort necessary to process slow subtitles.

If–as suggested by literature [2]–fast subtitles do not leave viewers sufficient time to watch the images, we might expect them to achieve low scores in the scene recognition test. It is also possible that with fast subtitles, viewers will not be able to read all the words in the subtitle and therefore miss some information from the dialogue, which will be evidenced by their being unable to recognize subtitles in the post-test questionnaire. If subtitles are too slow, however, viewers may end up re-reading them, possibly resulting in confusion, frustration and less enjoyment. In our study, following [58], we conceptualized enjoyment not only as “the sense of pleasure derived from consuming media products” [59], which relies mainly on the satisfaction of hedonic needs, but also on other, nonhedonic needs [60], including the satisfaction with basic subtitle parameters such as the optimum subtitle speed. We assumed that if viewers cannot keep up with fast subtitles, their enjoyment of the clip will be adversely affected.

When it comes to the differences between the videos in a language that is familiar (English in Exp. 2) and unfamiliar (Hungarian in Exp. 1) to viewers, we hypothesized that because people support their viewing with auditory information from the soundtrack, the preference for faster speeds and unreduced text may be more discernible when they understand the language of the film dialogue, whereas it may be less pronounced in the case of a language that viewers have no knowledge of. Furthermore, the analysis between different groups of subjects (Spanish, Polish and English) enabled us to consider the impact of experience with subtitling on the processing of subtitled videos. We expected that people who are familiar with subtitling may have developed certain strategies allowing them to process subtitles more efficiently, possibly evidenced by higher comprehension and lower cognitive load.

A powerful combination of different research methods: eye tracking, questionnaires and semi-structured interviews, has enabled us to isolate the impact of speeds on the processing of subtitled videos modulated by different linguistic backgrounds of viewers. To the best of our knowledge, no work to date has investigated subtitle speeds using such mixed methods approach. Our approach provides a unique research opportunity to determine whether modern viewers are able to keep up with fast subtitles and to measure their viewing experience in relation to different speeds, the language of the soundtrack and their familiarity with subtitling. Investigating these issues is particularly useful in the context of the multiplicity of subtitled content and current subtitling practices.

## Experiment 1

### Method

#### Participants

A total of 74 participants (aged 19–42, *M* = 26.55, *SD* = 5.86) took part in the study, of whom 27 were native speakers of English, 26 Spanish and 21 Polish (see Table 1). Participants were recruited from the UCL Psychology pool of volunteers, social media (Facebook page of the project, Twitter), and personal networking. They were pre-screened to be native speakers of English, Polish or Spanish, aged above 18 and to have no knowledge of Hungarian.

**Table 1:** Demographic information about participants.

Language				
		English	Polish	Spanish
Gender				
	Male	13	5	10
	Female	14	16	16
Age				
	Mean (SD)	27.59 (7.79)	24.71 (5.68)	28.12 (5.88)
	Range	20–54	19–38	19–42
AVT Preference				
	Subtitling	24	11	22
	Dubbing	0	0	1
	Voice-over	1	0	0
	Original version	1	10	3
	Doesn’t watch foreign films	1	0	0

Despite our expectations prior to the study and the linguistic background of the participants, when asked about the preferred type of audiovisual translation, the vast majority stated they prefer subtitling. This, on the one hand, may reflect changes in audiovisual translation landscape, and on the other may be attributed to the fact that the participants were living in the UK at the time the study was carried out. Finally, the preference for a given type of translation is not synonymous with its prevalence in a country; this is to say that although some participants may prefer subtitles now, they still grew up in a non-subtitling country.

The experiment was approved by the UCL Research Ethics Committee. Prior to testing, all participants received information sheets and provided written informed consent. In accordance with UCL hourly rates, each participant received £10 for taking part in the experiment.

#### Stimuli

Three self-contained dialogue-based clips, each lasting 4–6 minutes, from two films and one television series were used in the study: *Blue Jasmine* (2013, dir. Woody Allen), *Inside Out* (2015, dir. Pete Docter, Ronnie Del Carmen), and *Mad Men* (2007, creator: Matthew Weiner). All the three clips were fast-paced and dialogue-heavy, mostly featuring two to four characters engaged in a conversation. Each clip was subtitled at three different speeds: 12, 16 and 20 cps. Each participant watched three clips, one from each film. The order of presentation was counterbalanced following a Latin square design. Participants were allocated to one of the three groups: Group 1 saw *Blue Jasmine* subtitled at 20 cps, *Inside Out* at 16 cps and *Mad Men* at 12 cps; Group 2 saw *Blue Jasmine* subtitled at 16 cps, *Inside Out* at 12 cps and *Mad Men* at 20 cps; and Group 3 saw *Blue Jasmine* subtitled at 12 cps, *Inside Out* at 20 cps and *Mad Men* at 16 cps. The order of presentation of the clips in each group was randomized.

Subtitles were prepared in three language versions: English, Polish and Spanish, separately for each group of participants. The time codes (i.e., the subtitle display times) were identical for all the three languages–the translation was prepared in such a way that the number of characters in each version was carefully matched to the reading speed requirements, with 2-frames tolerance. Participants watched the clips with Hungarian audio and with subtitles in their mother tongue. All subtitle files are available in [61].

Below we present an English-version sample from *Blue Jasmine* (Table 2), showing the differences between the three conditions in terms of subtitle duration (to match exactly the reading speed requirements) and text condensation (if necessary).

**Table 2:** Example of slow, medium-paced and fast subtitles.

12 cps (slow)		16 cps (medium-paced)		20 cps (fast)	
Duration (ms)	Subtitle text	Duration (ms)	Subtitle text	Duration (ms)	Subtitle text
3320	I can't wait for you to show us New York.	2480	I can't wait for you to show us New York.	2040	I can't wait for you to show us New York.
1960	Take our car and driver!	1520	Take our car and driver!	1880	Why don't you take our car and driver?
3720	I hope you're gonna come. How often am I here?	2800	I hope you're gonna come. How often am I here?	2640	I hope you're gonna come. I mean, how often am I here?
2560	- I will make some time. - Good.	2600	- I will definitely make some time. - Good.	2120	- I will definitely make some time. - Good.
1800	Where are you staying?	1360	Where are you staying?	1120	Where are you staying?
4040	We thought about asking if we could stay with you,	3040	We thought about asking if we could stay with you,	2440	We thought about asking if we could stay with you,
2800	but we got a room at the Marriott.	3720	but we didn't want to impose. We got a room at the Marriott.	3080	but we didn't want to impose, so we got a room at the Marriott.

#### Procedure

Participants were tested individually in a lab. They were informed they would take part in a study about the quality of subtitles. The details related to the subtitle speed were only revealed during the post-test interview. Each experiment started with a training session, the results of which were not recorded, to familiarize the participants with the experimental procedure. The experiment, including all the instructions, subtitles and questions, was presented in English for English participants, in Polish for Polish participants and in Spanish for Spanish participants. Each experiment ended with a semi-structured interview to elicit viewers’ opinions and preferences on subtitle speeds.

#### Apparatus

An SMI RED 250 mobile eye tracker was used in the experiment. Participants’ eye movements were recorded with a sampling rate of 250Hz. The velocity-based saccade detection algorithm was used with the minimum duration of a fixation set to 80 ms. Participants with tracking ratio below 80% were excluded from the eye tracking analyses (but not from comprehension or self-reported cognitive load assessments). We used SMI software package Experiment Suite to create and conduct the experiment, and SPSS v. 24 to analyze the data.

#### Design

A 3x3 mixed ANOVA was used with subtitle speed (with three levels: 12, 16, 20 cps) as a within subject factor and native language (English, Spanish, Polish) as a between-subject factor.

#### Dependent variables

The dependent variables were: comprehension score, three indicators of self-reported cognitive load (difficulty, effort, frustration), enjoyment, scene recognition, subtitle recognition, and five eye tracking measures. We also collected categorical data on reading experience. Similarly to subtitles, all the questions were presented to the participants in their native languages.

#### Comprehension

Comprehension was measured after each clip as the percentage of correct answers to a set of multiple-choice and true/false questions related to the clip content. Below we present examples of multiple choice comprehension questions:

Example (1) from *Blue Jasmine*

What does Augie do for a living?

■ He does furniture moving and repairs
■ He is a musician and has his own band
■ He has his own company
■ I don’t know

Example (2) from *Inside Out*

What does Riley’s father threaten her with if she doesn’t eat her vegetables?

■ Not to let her go play hockey
■ Not to give her any dessert
■ Not to let her play with her best friend
■ I don’t know

and two examples of true/false questions:

Example (3) from *Blue Jasmine*

Ginger and Augie left the kids with Augie’s parents.

■ True
■ False

Example (4) from *Mad Men*

Dr Guttman told many people in the office about her report.

■ True
■ False

#### Self-reported cognitive load

Participants self-reported their cognitive load using three indicators: difficulty (related to the characteristics of the task: *Was it difficult for you to read the subtitles in this clip*?), effort (related to the participant’s mental work invested in following the task: *Did you have to put a lot of effort into reading the subtitles in this clip*?) and frustration (related to the participant’s feelings during the task: *Did you feel annoyed when reading the subtitles in this clip*?). They were assessed on 1–7 scale, where 1 meant “very low” and 7 “very high” cognitive load.

#### Enjoyment

Enjoyment was measured using the first item from Intrinsic Motivation Inventory [62]. The item was modified to reflect the nature of the task, focussing specifically on subtitles. Participants were asked to relate to the following statement: *I enjoyed watching the film with these subtitles*, using 1–7 scale, where 1 meant “not at all” and 7 means “very much”.

#### Scene recognition

After watching each clip, participants were presented with pairs of screenshots and asked to click on the one they had watched [61]. In each pair, both screenshots came from the film, but only one was taken from the scene presented to the participants (the left/right presentation order of screenshots was counterbalanced). The scene recognition variable was calculated as a percentage of correctly recognized scenes.

#### Subtitle recognition

Viewers were asked to recognize the phrasing from the subtitles in multiple choice questions in English, Polish and Spanish respectively (with three options: one correct answer, one distractor and *I don’t know*). The phrasing mostly differed at the end of the subtitle, the assumption being that if a subtitle is too fast, the viewer would not have time to read it until the end. Here is an English-language sample subtitle recognition question from *Mad Men*:

Example (5)

Which subtitle was used in the film?

■ I don’t see that in a TV commercial.
■ I don’t see that on a billboard.
■ I don’t know.

Subtitle recognition score was calculated as a percentage of correct answers.

#### Eye tracking

Table 3 presents the description of the eye tracking measures. We drew individual areas of interest (AOIs) on each subtitle in each clip (see .xml files with AOIs available in [61]). The results reported here are averaged per clip and per participant.

**Table 3:** Definitions of the eye tracking measures.

Eye tracking measure	Description
Fixation count	The number of fixations in the AOI, averaged per participant per clip. High fixation count can be indicative of higher cognitive effort and/or poorer reading skills.
Mean fixation duration	The duration of a fixation in a subtitle AOI, averaged per clip and per participant. Longer mean fixation duration may be an indication of higher cognitive load.
Absolute reading time	Dwell time, i.e., the sum of durations of all fixations and saccades in an AOI starting with the first fixation. Longer reading time may be related to processing difficulties.
Proportional reading time	The percentage of dwell time a participant spent in the AOI as a function of subtitle display time. For example, if a subtitle lasted for 3 seconds and the participant spent 2.5 seconds in that subtitle, the percentage dwell time was 2500/3000 ms = 83%, i.e. while the subtitle was displayed for 3 seconds, the participant was looking at that subtitle for 83% of the time. This measure allowed us to compare the proportion of time spent in the subtitles between different conditions. If proportional reading time approaches 100%, it means that participants spent most of their time reading subtitles and did not have time to look at on-screen action.
Revisits	The number of glances a participant made to the subtitle AOI after visiting the subtitle for the first time. Revisits may indicate a more disruptive reading process, as the participant goes back to read the AOI they had already read, for instance because of text difficulty or because the subtitle is displayed for too long.

#### Reading experience

To assess how participants coped with different subtitle speeds, we asked them if they had enough time to read the subtitles, if they re-read subtitles, if they missed words from the subtitles, if they had enough time to read the subtitles and follow the on-screen action and, finally, what subtitles they prefer: verbatim or condensed.

All the questionnaires, results and raw data from both experiments are available in the Repository for Open Data (RepOD) curated at the University of Warsaw [61].

### Results

#### Comprehension

Subtitle speed did not have an effect on comprehension, *F*(2,140) = 0.114, *p =* .892, $\eta_{p}^{2}$ = .002 (see Table 4 for descriptive statistics). There were no interactions.

**Table 4:** Percentage of correct comprehension answers in Experiment 1.

	Subtitle speed		
Language	12 cps	16 cps	20 cps
	*M (SD)*	*M (SD)*	*M (SD)*
English	78.40 (17.75)	79.48 (18.17)	79.85 (15.97)
Polish	88.13 (10.55)	85.09 (13.08)	85.77 (11.75)
Spanish	73.00 (14.95)	77.53 (17.71)	76.93 (12.38)
Total	79.35 (16.00)	80.42 (16.76)	80.55 (13.94)

We found a main effect of language on comprehension, *F*(2,70) = 6.07, *p* = .004, $\eta_{p}^{2}$ = .148. Post-hoc comparisons with Bonferroni correction showed that Polish participants achieved a higher comprehension score compared to the Spanish (*p* = .003, 95% CI [3.01, 18.01]); they also had a tendency to have higher comprehension than the English (*p* = .063, 95% CI [-0.28, 14.45]). English and Spanish participants did not differ from each other, *p* = .707, 95% CI [-3.6, 10.45].

#### Self-reported cognitive load

Expecting that fast subtitles would be more difficult, effortful and frustrating for viewers to process, we asked the participants to assess the difficulty of the subtitles in the clips as well as the effort they had to expend in watching them, and the level of frustration they experienced. We found the main effect of speed on difficulty and effort but not on frustration (see Table 5). Participants generally declared the lowest cognitive load in the case of slow subtitles (12 cps). There were no interactions.

**Table 5:** Mean cognitive load indicators in Experiment 1.

	Subtitle speed						
	12	16	20	*df*	*F*	*p*	$\eta_{p}^{2}$
	*M (SD)*	*M (SD)*	*M (SD)*				
Difficulty				2,140	4.329	.015*	.058
English	2.41 (1.44)	2.85 (1.43)	2.81 (1.77)				
Polish	1.52 (1.16)	2.24 (1.60)	2.14 (1.42)				
Spanish	1.64 (1.25)	2.20 (1.55)	2.12 (1.42)				
Effort				2,140	3.484	.033*	.047
English	3.33 (1.68)	3.63 (1.73)	3.56 (2.15)				
Polish	1.90 (1.22)	2.10 (1.30)	2.33 (1.31)				
Spanish	1.56 (.71)	2.28 (1.64)	2.44 (1.38)				
Frustration				2,140	1.811	.167	.025
English	2.59 (1.52)	2.59 (1.44)	2.70 (1.61)				
Polish	1.57 (1.20)	1.48 (.92)	2.00 (1.34)				
Spanish	1.52 (1.26)	2.24 (1.71)	2.08 (1.22)				

*Note*. Effort is reported with Greenhouse-Geisser correction as sphericity assumption was violated (Maunchly’s Test of Sphericity, *p* = .003).

There was a main effect of language on all the three indicators of cognitive load (see Table 6). Post-hoc Bonferroni tests showed that in difficulty, English participants declared highest cognitive load, which differed from that declared by the Polish participants (*p* = .058, 95% CI [-0.2, 1.46]) and by the Spanish (*p =* .051, 95% CI [.00, 1.41]). In effort, English participants also had higher load compared to Polish (*p* < .000, 95% CI [0.56, 2.23]) and to Spanish (*p* < .000, 95% CI [0.62, 2.21]). Similarly, English participants reported higher frustration compared to Polish (*p =* .007, 95% CI [0.21, 1.68]) and to Spanish (*p =* .057, 95% CI [-0.2, 1.38]). Polish and Spanish participants did not differ from each other in any of the indicators.

**Table 6:** Between-subjects effects in cognitive load in Experiment 1.

Cognitive load indicator	df	*F*	*P*	$\eta_{p}^{2}$
Difficulty	2,70	4.018	.022*	.103
Effort	2,70	12.378	.000*	.261
Frustration	2,70	5.598	.006*	.138

#### Enjoyment

Predicting that subtitles that are too slow or too fast may negatively affect viewers’ enjoyment, we asked the participants to assess their enjoyment of the clips. Despite our predictions, however, subtitle speed did not affect enjoyment (see Table 7), nor did the language, *F*(2,70) = 1.108, *p =* .336, $\eta_{p}^{2}$ = .031, which shows that participants enjoyed the clips equally, regardless of their native language and subtitle speed.

**Table 7:** Mean enjoyment in Experiment 1.

	Subtitle speed						
	12	16	20	*df*	*F*	*p*	$\eta_{p}^{2}$
	*M (SD)*	*M (SD)*	*M (SD)*				
Enjoyment				2,140	.391	.677	.006
English	5.15 (1.35)	4.96 (1.55)	4.93 (1.73)				
Polish	5.24 (1.44)	5.33 (1.39)	4.90 (1.33)				
Spanish	5.36 (1.57)	5.52 (1.58)	5.48 (1.22)				

However, following the reviewers’ suggestions, we also examined potential differences in enjoyment depending on the films genres, predicting that viewers may simply have enjoyed certain films more than others independently of the subtitle speed. Indeed, we found a main effect of film on enjoyment, *F*(2, 140) = 25.196, *p* < .000, $\eta_{p}^{2}$ = .265. The highest enjoyment was declared by participants in the case of the cartoon *Inside Out* (*M* = 5.89, *SD* = 1.23), followed by *Blue Jasmine* (*M* = 5.07, *SD* = 1.35) and the lowest enjoyment level was found in *Mad Men* (*M* = 4.66, *SD* = 1.54). There was no effect of language, *F*(2, 70) = 1.108, p = .336, $\eta_{p}^{2}$ = .031, which means that all participants enjoyed the films similarly regardless of their mother tongue.

#### Scene recognition

To verify whether fast subtitles hinder viewers’ ability to follow on-screen action, we administered a scene recognition test. Contrary to expectations, scene recognition was not affected by speed, *F*(2,140) = .038, *p* = .963, $\eta_{p}^{2}$ = .001 (see Table 8 for descriptive statistics), or by language, *F*(2,70) = 1.707, *p* = .189, $\eta_{p}^{2}$ = .046. This means that all groups of participants could recognize the scenes equally well in all speed conditions.

**Table 8:** Percentage of correct scene recognition questions in Experiment 1.

	Subtitle speed		
Language	12 cps	16 cps	20 cps
	*M (SD)*	*M (SD)*	*M (SD)*
English	97.40 (7.12)	98.88 (4.23)	97.77 (8.47)
Polish	99.04 (4.36)	100.00 (.00)	99.52 (2.18)
Spanish	99.60 (2.00)	96.40 (8.48)	98.20 (5.56)
Total	98.63 (5.08)	98.35 (5.71)	98.42 (6.17)

#### Subtitle recognition

Based on the assumption that if subtitles are too fast, viewers may not be able to read them, we asked the participants to recognize phrases from subtitles. We predicted that if people did not manage to read a subtitle in its entirety, their ability to recognize the subtitle wording would be hampered. However, the impact of speed on subtitle recognition did not reach statistical significance, *F*(2,140) = 2.529, *p* = .083, $\eta_{p}^{2}$ = .035 (see Table 9). There were no interactions.

**Table 9:** Percentage of correct subtitle recognition in Experiment 1.

	Subtitle speed		
Language	12 cps	16 cps	20 cps
	*M (SD)*	*M (SD)*	*M (SD)*
English	84.44 (15.30)	73.08 (22.32)	77.40 (24.77)
Polish	72.38 (22.58)	70.63 (22.59)	80.15 (22.24)
Spanish	69.20 (25.04)	63.33 (24.28)	65.46 (23.48)
Total	75.75 (21.95)	69.04 (23.16)	74.10 (24.16)

We found a main effect of language on subtitle recognition, *F*(2,70) = 3.458, *p* = .037, $\eta_{p}^{2}$ = .090. Post-hoc Bonferroni tests showed that English participants had a significantly higher subtitle recognition compared to Spanish, *p* = .035, 95% CI [0.65, 23.97], who in general had the lowest scores. Polish and English participants did not differ significantly.

#### Eye tracking measures

Owing to poor quality, some data had to be removed, leaving 22 English, 16 Polish, and 22 Spanish participants included in the eye tracking analyses.

**Fig 1:**
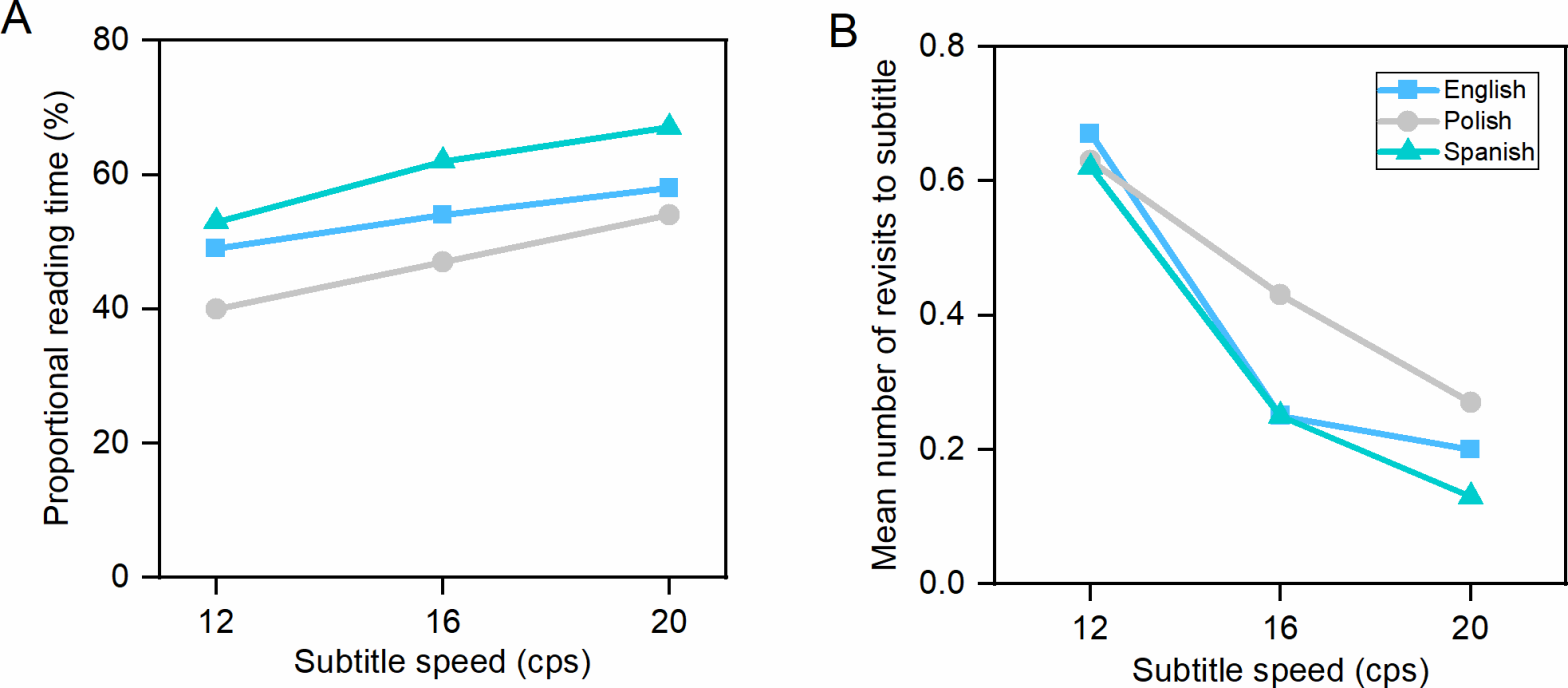
Reading measures in clips with an unknown language soundtrack (Hungarian). (A) Proportional reading time (percentage of time spent in the subtitle relative to the total subtitle display time). (B) Mean number of revisits to the subtitle.

Subtitle speed had an effect on all eye tracking measures (Table 10). There were no interactions. Slower subtitles induced more fixations and higher mean fixation duration than faster subtitles. The absolute reading time was longest in the 12 cps condition, whereas the proportional reading time was highest in the 20 cps condition. Fig 1A shows that an increase in subtitle speeds resulted in an increase in the percentage of time spent in the subtitle area, relative to subtitle duration. Subtitles in the slowest condition (12 cps) triggered the largest number of revisits, which may mean that participants read the subtitle, looked at the scene and gazed back at the subtitle area, only to find the same subtitle there. We discovered a trend, depicted in Fig 1B, that the longer the subtitle duration, the more revisits to the subtitle area. When watching slow subtitles, viewers re-read two out of three subtitles, but when watching fast subtitles, they re-read about one in five.

**Table 10:** Eye tracking measures in Experiment 1.

	Subtitle speed						
	12	16	20	*df*	*F*	*P*	$\eta_{p}^{2}$
Fixation count				2,114	10.752	.000*	.159
English	6.60	5.64	5.32				
Polish	5.97	5.62	5.08				
Spanish	6.67	6.06	5.91				
Mean fixation duration				2,114	9.098	.000*	.138
English	206	196	191				
Polish	180	175	176				
Spanish	217	210	204				
Absolute reading time				2,114	11.719	.000*	.171
English	1554	1265	1191				
Polish	1243	1146	1070				
Spanish	1625	1443	1364				
Proportional reading time				2,114	65.356	.000*	.534
English	.49	.54	.58				
Polish	.40	.47	.54				
Spanish	.53	.62	.67				
Revisits				2,114	89.533	.000*	.611
English	.67	.25	.20				
Polish	.63	.43	.27				
Spanish	.62	.25	.13				

*Note*. Mean fixation duration and absolute reading time measures are given in milliseconds.

There was a main effect of language on all dependent variables apart from revisits (Table 11). Post-hoc Bonferroni tests showed that Polish participants spent the least amount of time in the subtitles and differed significantly from Spanish participants in all eye tracking measures (fixation count, *p =* .029, 95% CI [-1.26, -.05]; mean fixation duration, *p =* .001, 95% CI [-56.23, -11.34]; absolute reading time, *p* < .000, 95% CI [-491.85, -156.68] and proportional reading time, *p* < .000, 95% CI [-.20, -.06]) and from English participants in the case of absolute reading time (*p =* .027, 95% CI [-351.12, -15.96]). Overall, Spanish participants dwelled longest in the subtitle area and their fixation duration was the longest, indicating highest effort among all the groups.

**Table 11:** Eye tracking results for between-subjects effects in Experiment 1.

Eye tracking measure	df	*F*	*p*	$\eta_{p}^{2}$
Absolute reading time	2,57	11.393	.000*	.286
Fixation count	2,57	3.675	.032*	.114
Revisits	2,57	1.740	.185	.058
Mean fixation duration	2,57	6.909	.002*	.195
Proportional reading time	2,57	10.266	.000*	.265

#### Reading experience

**Fig 2:**
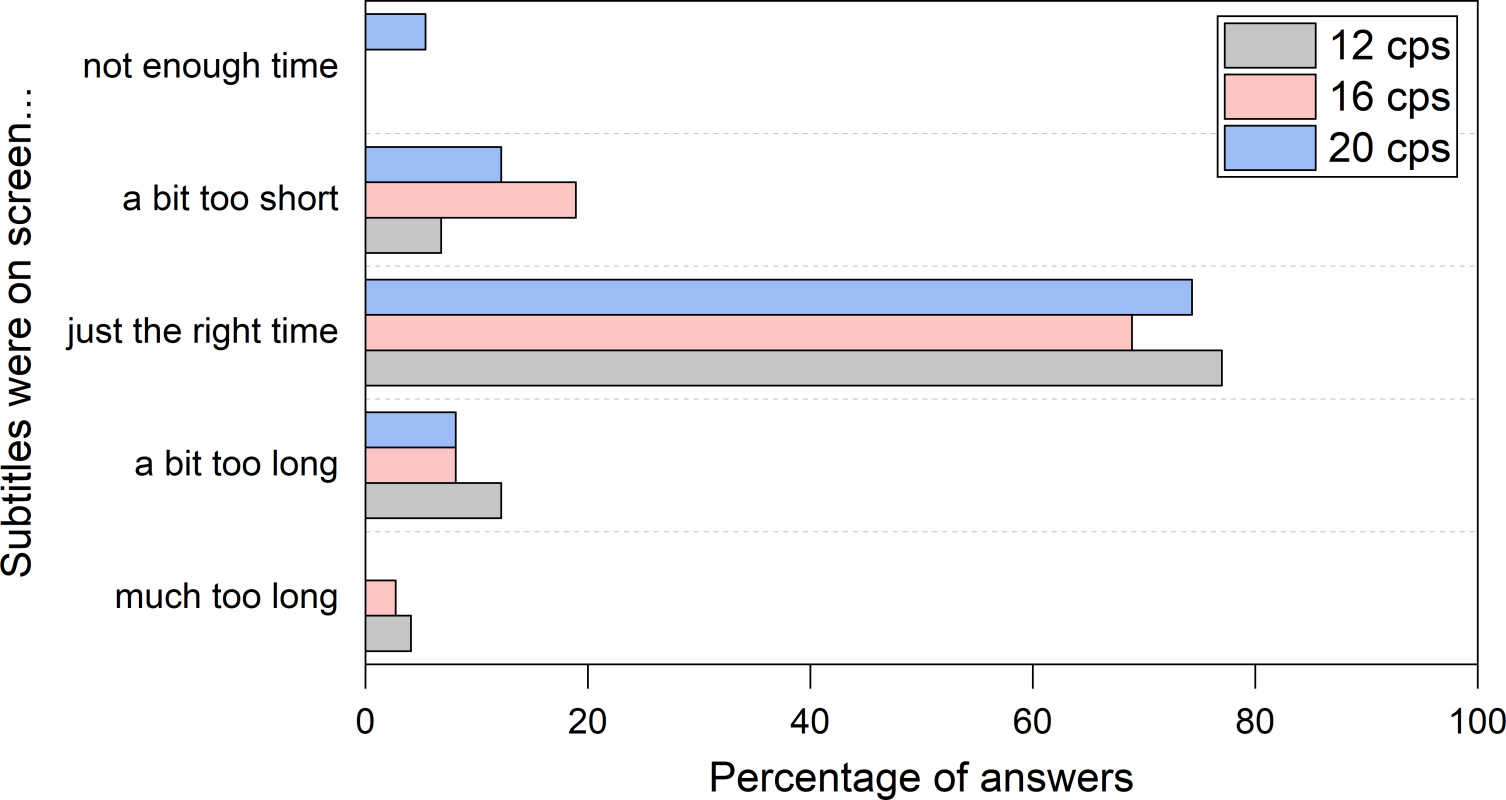
Perceived subtitle duration in clips with an unknown language soundtrack (Hungarian).

**Fig 3:**
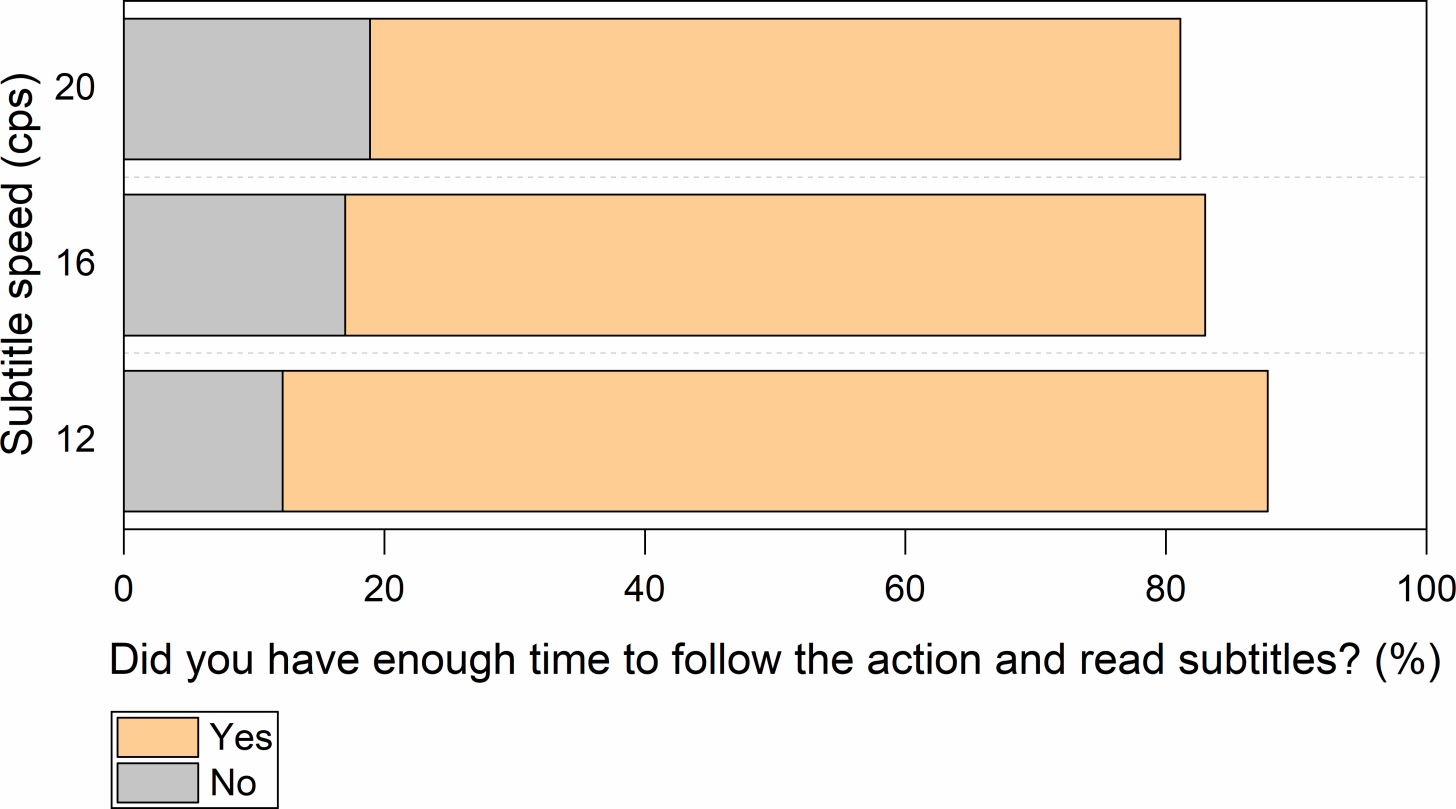
Perceived time to follow the action in clips with an unknown language soundtrack (Hungarian). Participants had to choose one of two options: (1) *I had enough time to read the subtitles and follow the action on screen*, (2) *I didn’t have enough time to follow the action on screen as I was reading the subtitles*.

Reading experience questions showed that participants in general could cope well with all the three speeds. Most participants declared that subtitles were displayed for the right amount of time (Fig 2) and that they had sufficient time to read them as well as to follow the on-screen action (Fig 3).

Most people also declared that when reading the subtitles, they did not miss words (Table 12). They also stated that they re-read subtitles more often in the slow subtitles condition (12 cps) compared to the fast subtitles. When asked about text condensation in subtitles, most people–regardless of subtitle speed–declared they would prefer subtitles to contain less text so that they have more time to read them.

**Table 12:** Self-reported reading experience in clips in Hungarian.

	12 cps	16 cps	20 cps
When watching this clip…			
I missed many words	0	0	1.4
I missed some of the words	12.2	13.5	10.8
I didn’t miss many words	21.6	28.4	31.1
I didn’t miss any words	66.2	56.8	56.8
Re-reading			
I re-read many subtitles	2.7	5.4	1.4
I re-read some of the subtitles	31.1	21.6	16.2
I generally didn’t re-read the subtitles	40.5	45.9	44.6
I didn’t re-read any of the subtitles	25.7	25.7	37.8
Preferences			
I prefer subtitles to contain less text so that I have more time to read them	58.1	62.2	63.5
I prefer subtitles to contain as much dialogue as possible, even if it means they appear and disappear very fast	41.9	36.5	36.5

*Note*. The data are given as a percentage of the people who selected a given answer

#### Correlations

In order to gain insights into participants’ cognitive load experienced when watching clips subtitled at different speeds, we correlated the results of self-reports with eye tracking data. Using Spearman’s rank correlation, we found that in the 12 cps condition, there was a significant correlation between the self-reported difficulty and revisits to the subtitle: *r*_*s*_(64) = .352, *p* = .004, and between the self-reported effort and absolute reading time, *r*_*s*_(64) = .253, *p* = .043. This means that the more revisits participants made to the slow subtitles and the longer they read them, the more cognitive effort they reported. Self-reported difficulty was also negatively related to comprehension, *r*_*s*_(74) = -.331, *p* = .004, and to enjoyment, *r*_*s*_(74) = -.441, *p* < .000, indicating that the more difficult the participants found the clip, the lower their comprehension score and enjoyment. In the 16 cps condition, the self-reported difficulty was significantly related to the absolute reading time, *r*_*s*_(63) = .300, *p* = .017, and to fixation count, *r*_*s*_(63) = .282, *p* = .025. Self-reported effort was also related to absolute reading time, *r*_*s*_(63) = .343, *p* = .006, and to fixation count, *r*_*s*_(63) = .317, *p* = .011. The more difficult the participants found the clip, the more time they spent reading the subtitles. At the same time, the more time they spent reading the subtitles, as shown by their absolute reading time, the less enjoyment they reported, *r*_*s*_(63) = -.403, *p* = .001, and the lower their comprehension score, *r*_*s*_(63) = -.709. Enjoyment was also negatively correlated with revisits: the more revisits the participants made, the lower enjoyment they reported, *r*_*s*_(63) = -.327, p = .009. There was also a significant relationship between self-reported frustration and mean fixation duration, *r*_*s*_(63) = .297, *p* = .018. Finally, in the 20 cps condition, enjoyment was negatively correlated with all indicators of self-reported cognitive load: difficulty, *r*_*s*_(74) = -.457, *p* < .000; effort, *r*_*s*_(74) = -.412, *p* < .000; and frustration, *r*_*s*_(74) = -.508, *p* < .000, showing that the more cognitive effort the participants expended, the less enjoyment they reported.

#### Discussion

Contrary to our expectations, subtitle speed did not affect comprehension, scene recognition, or enjoyment. As demonstrated by reading experience questions, participants were equally satisfied with the speed of subtitles in the slow, medium and fast conditions. In all speeds, participants declared to have had sufficient time to read the subtitles and to follow the on-screen action.

The slowest subtitles displayed at 12 cps induced most re-reading, as shown by the highest number of revisits to the subtitle area in eye tracking data as well as by participants’ own declarations on re-reading. At the same time, however, the slowest subtitles were deemed the least difficult and effortful to read.

Overall, the highest cognitive load was reported by English participants, who are generally not accustomed to subtitling. In contrast, Polish participants declared lowest frustration among all the three groups. The self-report results were confirmed by eye tracking as they also spent the least amount of time in the subtitles and had lowest mean fixation duration, indicating lowest cognitive load. Despite the results from the self-reported cognitive load and enjoyment, eye tracking data showed that Spanish participants may have experienced higher cognitive load as they had highest mean fixation duration and spent the highest time reading the subtitles, as shown by both the absolute and proportional reading time. Their comprehension results were also the lowest.

## Experiment 2

### Method

#### Participants

For Experiment 2, Polish and Spanish speakers self-reported their proficiency in listening to English using the Common European Framework of Reference for Languages (from A1 to C2). Among Polish participants, 4 declared to be at the C1 and 17 at C2 level, whereas for Spanish, 1 person said to be A2, 2 people at B1, 3 people at B2, 7 people at C1 and 13 people at C2 level. We need to note that the English proficiency of our sample was high as at the time the study was taking place, participants were living in the UK. Given their high proficiency, with most participants at the C1 and C2 levels, we could not analyze the data using English proficiency as a factor, as we simply did not have enough participants with limited knowledge of English. We acknowledge this as a limitation of the study.

#### Stimuli

In this experiment, for clarity reasons, only two subtitle speeds were tested: slow (12 cps) and fast (20 cps). A set of two clips in English, lasting approximately 5 minutes each, from two TV series was used, one from *Gilmore Girls* (2000, created by Amy Sherman-Palladino), the other from *Grace and Frankie* (2015, created by Marta Kauffman and Howard J. Morris). As in Exp. 1, the stimuli were fast-paced, dialogue-heavy scenes featuring two to four characters engaged in a conversation.

Similarly to Experiment 1, subtitles were prepared in three language versions: English, Polish and Spanish, separately for each group of participants. The time codes were identical for all the languages. We used Latin square design and divided participants into two groups: Group 1 saw *Gilmore Girls* subtitled at 20 cps and *Grace and Frankie* at 12 cps, whereas Group 2 saw *Gilmore Girls* subtitles at 12 cps and *Grace and Frankie* at 20 cps. Unlike in Experiment 1, this time the clips were shown with their original English soundtrack.

#### Design

A 2x3 mixed ANOVA was used with subtitle speed (with 2 levels: 12, 20 cps) as a within subject factor, and first language (English, Spanish, Polish) as a between-subject factor.

The dependent variables were the same as in Exp. 1. Because in this experiment the participants were familiar with the language of the soundtrack, we wanted to see if they noticed the edits made to subtitle text in slower subtitles. For this reason, we added the measure of *Perceived Mismatch* between the dialogue and the subtitles (*Did you notice any mismatch between the spoken dialogue and the subtitles*?), reported on 1–7 scale, where 1 meant “no mismatch” and 7 “a lot of mismatches”.

### Results

#### Comprehension

Similarly to Experiment 1, we found no main effect of subtitle speed on comprehension, *F*(1,66) = .012, *p =* .913, $\eta_{p}^{2}$ = .000. There was no main effect of language on comprehension, *F*(2,66) = .080, *p =* .923, $\eta_{p}^{2}$ = .002. There were no interactions. Descriptive results are depicted in Table 13.

**Table 13:** Percentage of correct comprehension answers in Experiment 2.

	Subtitle speed	
Language	12 cps *M (SD)*	20 cps *M (SD)*
English	85.57 (15.22)	89.87 (11.97)
Polish	89.86 (12.12)	87.40 (12.32)
Spanish	89.59 (9.62)	88.22 (11.54)
Total	88.39 (12.35)	88.50 (1178)

#### Self-reported cognitive load

There was no main effect of subtitle speed on difficulty or effort, but we found a significant effect of speed on frustration (see Table 14). Frustration was lower in the 20 cps condition for all groups of participants.

**Table 14:** Mean cognitive load indicators in Experiment 2.

	Subtitle speed					
	12	20	*df*	*F*	*p*	$\eta_{p}^{2}$
	*M (SD)*	*M (SD)*				
Difficulty			1,71	.164	.687	.002
English	2.30 (1.89)	1.89 (1.28)				
Polish	1.33 (.48)	1.95 (1.32)				
Spanish	1.62 (1.13)	1.62 (1.09)				
Effort			1,71	.306	.582	.004
English	2.85 (1.91)	2.22 (1.57)				
Polish	1.38 (.74)	2.33 (1.49)				
Spanish	1.62 (.75)	1.58 (.70)				
Frustration			1,71	14.559	.000*	.170
English	3.63 (1.90)	2.15 (1.13)				
Polish	1.86 (1.65)	1.57 (.87)				
Spanish	2.00 (1.64)	1.65 (1.23)				

We also found an interaction between speed and language in effort, *F*(2,71) = 6.935, *p =* .002, $\eta_{p}^{2}$ = 163) and in frustration, *F*(2,71) = 4.658, *p =* .013, $\eta_{p}^{2}$ = .116). We decomposed these interactions with simple effects with Bonferroni correction and found a main effect of subtitle speed on frustration in the English, *F*(1,26) = 16.980, *p =* .000, $\eta_{p}^{2}$ = .395, and Spanish group, *F*(1,25) = 4.355, *p =* .047, $\eta_{p}^{2}$ = .148. Frustration was lower in the 20 cps condition compared to 12 cps. For Polish speakers, there was a main effect of subtitle speed on effort, *F*(1,20) = 14.134, *p =* .001, $\eta_{p}^{2}$ = .414 but not for frustration. Polish participants declared to expend more effort when reading faster subtitles displayed at 20 cps compared to the slow subtitles.

We also found a main effect of language on effort, *F*(2,71) = 12.442, *p =* .007, $\eta_{p}^{2}$ = .130, and on frustration, *F*(2,71) = 7.060, *p =* .002, $\eta_{p}^{2}$ = .166. English participants experienced highest frustration than other groups. Post-hoc tests (Bonferroni) showed that in the case of effort, English participants differed from Spanish (*p* = .007, 95% CI [0.21, 1.67]) and in the case of frustration, they differed from both Spanish (*p =* .007, 95% CI [0.23, 1.89]) and from Polish participants (*p =* .005, 95% CI [0.3, 2.05]). Spanish and Polish participants did not differ from each other.

#### Enjoyment

Unlike in Experiment 1, we found a main effect of speed on enjoyment and no main effect of language on enjoyment, *F*(2,71) = .857, *p* = .429, $\eta_{p}^{2}$ = .024. There was an interaction between speed and language approaching significance, *F*(2,71) = 2.809, *p* = .067, $\eta_{p}^{2}$ = .073. For English and Spanish participants, enjoyment was higher for the faster subtitle speed, while for the Polish participants enjoyment was the same for both speeds (see Table 15).

**Table 15:** Enjoyment in Experiment 2.

	Subtitle speed					
	12	20	*df*	*F*	*p*	$\eta_{p}^{2}$
	*M (SD)*	*M (SD)*				
Enjoyment			1,71	8.893	.004*	.111
English	4.26 (1.73)	5.44 (1.36)				
Polish	5.10 (1.84)	5.10 (1.37)				
Spanish	5.00 (1.6)	5.62 (1.23)				

Similarly to Exp. 1, we wanted to see whether enjoyment declared by participants depended on the film itself. In contrast to Exp. 1, we did not find a main effect of film on enjoyment, *F*(1,71) = 2.784, *p* = .100, $\eta_{p}^{2}$ = .038. Polish and English participants enjoyed *Grace and Frankie* (*M*_*Pol*_ = 5.52, *SD* = 1.24; *M*_*Eng*_ = 5.00, *SD* = 1.68) slightly more than *Gilmore Girls* (*M*_*Pol*_ = 4.66, *SD* = 1.82; *M*_*Eng*_ = 4.70, *SD* = 1.63), whereas Spanish participants enjoyed both clips nearly equally (*M*_*GG*_ = 5.34, SD = 1.32; *M*_*GF*_ = 5.26, *SD* = 1.58), but the differences did not reach significance.

#### Perceived mismatch

**Fig 4:**
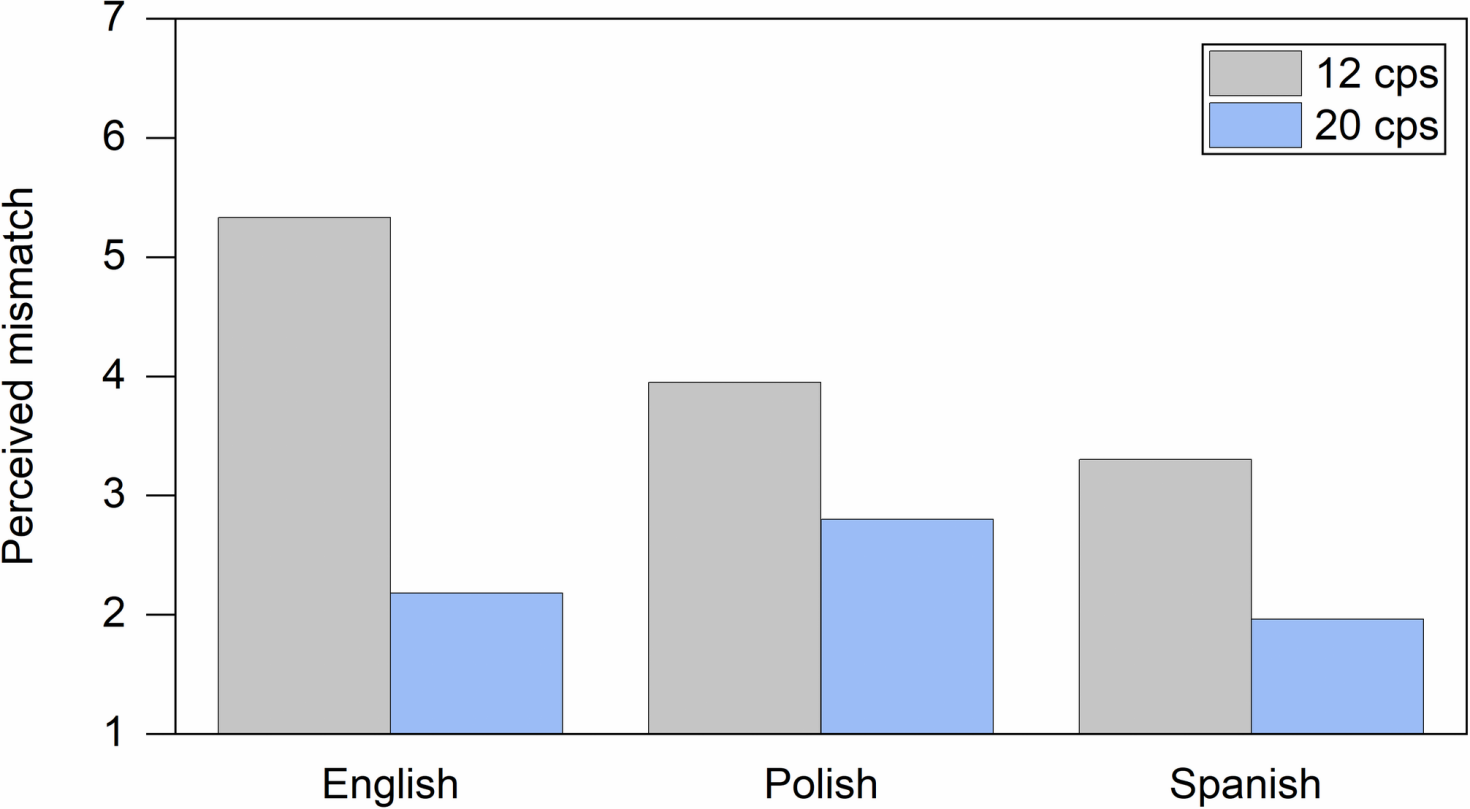
Perceived mismatch in clips with a known language soundtrack (English). Self-report on a scale 1–7, where (1) meant “no mismatch” and (7) “a lot of mismatches”.

Given that the participants could understand the language of the soundtrack and given the differences in subtitled content resulting from text editing in the two speed conditions, we expected participants to notice discrepancies between the spoken dialogues and the text in the slow subtitles. Indeed, we found a strong main effect of the speed on the degree of perceived mismatch between the dialogue and the text in the subtitles, *F*(1,71) = 84.115, *p <* .000, $\eta_{p}^{2}$ = .542. Slow subtitles were declared to have a considerably higher degree of mismatch (*M*_*12*_ = 4.22, *SD* = 1.95) than fast subtitles (*M*_*20*_ = 2.28, *SD* = 1.31) by all groups of participants (see Fig 4). The median values were 4.50 for the 12 cps condition and 2.00 for the 20 cps condition.

We found a significant effect of language on perceived mismatch, *F*(2,71) = 5.269, *p =* .007, $\eta_{p}^{2}$ = .129. English participants noticed more mismatches than Polish and Spanish people, but only with the Spanish was the difference significant, *p =* .006, 95% CI [0.26, 1.98]. This result is not surprising given that English participants were watching clips in their mother tongue.

#### Scene recognition

There was no main effect of speed on scene recognition (see Table 16), but we found a main effect of language, *F*(2,71) = 26.742, *p* < .000, $\eta_{p}^{2}$ = .430. Post-hoc tests with Bonferroni correction showed a significant difference between English participants on the one hand and Polish and Spanish on the other hand, *ps* < .000. English participants had a significantly lower scene recognition score than Polish participants, 95% CI [-24.25, -10.71], and Spanish, 95% CI [-22.51, -9.72], suggesting they focused more on comparing the subtitles with the dialogues rather than on the visuals.

**Table 16:** Percentage of scenes recognized correctly in Experiment 2.

	Subtitle speed					
	12	20	*df*	*F*	*p*	$\eta_{p}^{2}$
	*M (SD)*	*M (SD)*				
Scene recognition			1,71	.142	.707	.002
English	82.53 (24.49)	78.41 (24.06)				
Polish	97.95 (5.12)	97.95 (5.12)				
Spanish	96.15 (7.62)	97.03 (6.28)				

#### Subtitle recognition

While speed did not affect subtitle recognition (see Table 17), a significant interaction was found between speed and language, *F*(2,71) = 4.096, *p =* .021, $\eta_{p}^{2}$ = .103. There was a main effect of speed in the case of English participants *F*(1,26) = 5.603, *p =* .026, $\eta_{p}^{2}$ = .177 but not in the case of Polish, *F*(1,20) = .604, *p =* .446, $\eta_{p}^{2}$ = .029, or Spanish participants, *F*(1,25) = 1.997, *p =* .170, $\eta_{p}^{2}$ = .074. For English participants, subtitle recognition was higher in the 20 cps condition compared to 12 cps, whereas for Polish and Spanish people it was slightly lower for the 20 cps subtitles.

**Table 17:** Percentage of subtitles recognized correctly in Experiment 2.

	Subtitle speed					
	12	20	*df*	*F*	*p*	$\eta_{p}^{2}$
	*M (SD)*	*M (SD)*				
Subtitle recognition			1,71	.002	.965	.000
English	85.8 (21.03)	95.06 (7.75)				
Polish	86.5 (15.47)	83.33 (13.94)				
Spanish	80.12 (18.26)	73.71 (22.19)				

There was a significant main effect of language on subtitle recognition, *F*(2,71) = 6.443, *p =* .003, $\eta_{p}^{2}$ = .154. English participants recognized a significantly higher number of scenes than Spanish participants, *p =* .002, 95% CI [4.24, 22.77].

#### Eye tracking measures

Due to data quality issues, several participants had to be excluded from eye tracking analyses, leaving a total of 23 English, 19 Polish, and 22 Spanish participants.

Similarly to Experiment 1, we found the main effect of subtitle speed on all eye tracking measures (see Table 18). The slow subtitles induced more fixations than the fast ones. In all groups of participants, the mean fixation duration was lower in the 20 cps condition. Absolute reading time for the 20 cps condition was lower than the 12 cps condition. Proportional reading time, however, was higher for faster subtitles.

**Table 18:** Mean eye tracking measures in Experiment 2.

	Subtitle speed					
	12	20	*df*	*F*	*p*	$\eta_{p}^{2}$
Fixation count			1,61	56,105	,000*	,479
English	5.04	3.84				
Polish	5.40	4.24				
Spanish	6.01	4.63				
Mean fixation duration			1,61	9,252	,003*	,132
English	218	199				
Polish	188	180				
Spanish	213	210				
Absolute reading time			1,61	46,458	,000*	,432
English	1212	848				
Polish	1151	877				
Spanish	1416	1084				
Proportional reading time			1,61	26,378	,000*	,302
English	.40	.44				
Polish	.39	.46				
Spanish	.48	.58				
Revisits			1,61	224,926	,000*	,787
English	.50	.19				
Polish	.60	.18				
Spanish	.61	.16				

*Note*. Mean fixation duration and absolute reading time measures are given in milliseconds.

**Fig 5:**
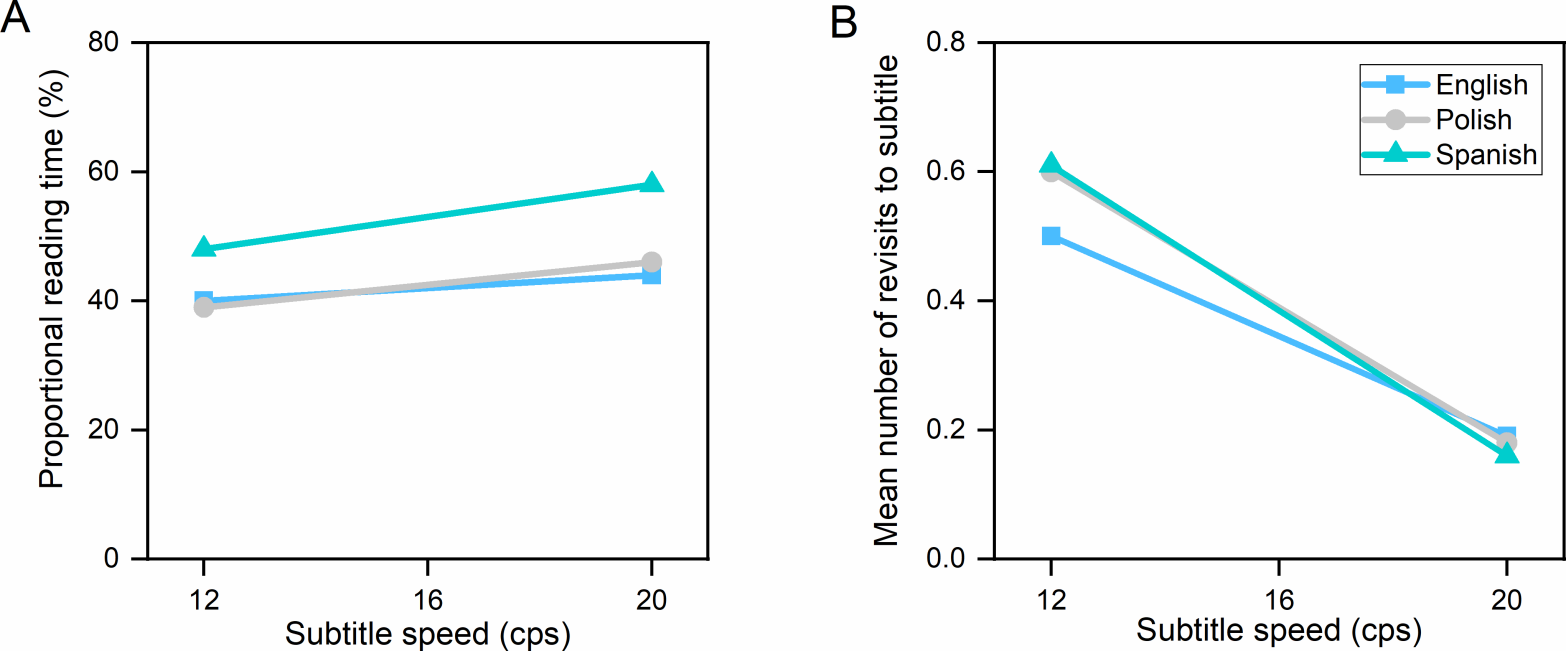
Reading measures in clips with a known language soundtrack (English). (A) Proportional reading time (percentage of time spent in the subtitle relative to the total subtitle display time). (B) Mean number of revisits to the subtitle.

There was an interaction between speed and language in the case of revisits, *F*(2,61) = 3.172, *p* = .049, $\eta_{p}^{2}$ = .094, which we decomposed with simple effects using Bonferroni correction. There was a main effect of speed on revisits in the English group, *F*(1,22) = 47,039, *p* < .000, $\eta_{p}^{2}$ = .681, Polish group, *F*(1,18) = 102.364, *p* < .000, $\eta_{p}^{2}$ = .850, and Spanish group, *F*(1,21) = 89.796, *p* < .000, $\eta_{p}^{2}$ = .810. English people had the lowest number of revisits in the 12 cps condition and the higher number of revisits in the 20 cps condition compared to Polish and Spanish people (see. Fig 5).

The implication of the number of revisits to the subtitle area for the subtitle reading process is that when watching slow subtitles, viewers re-read every second subtitle, whereas in the case of the fast subtitles, only one in five or one in six was re-read. This may be taken to mean that slow subtitles resulted in a more disrupted reading process.

**Fig 6:**
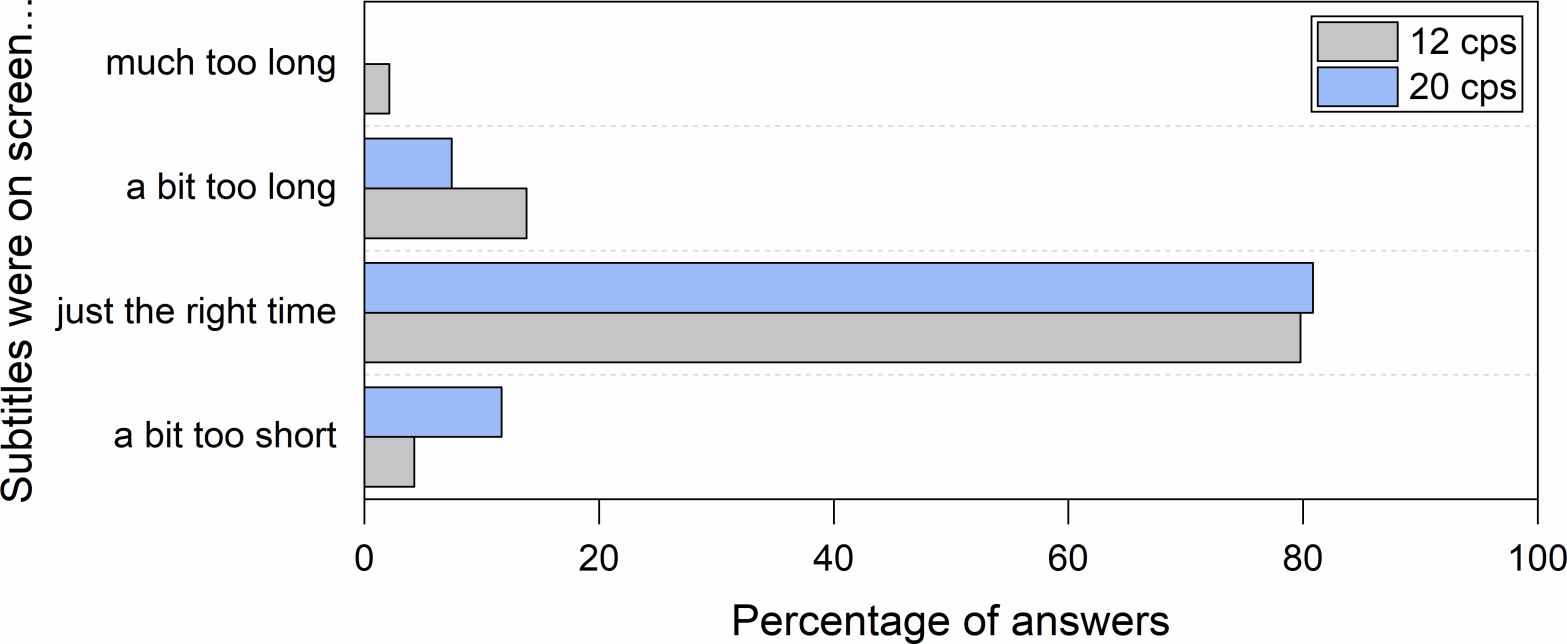
Perceived subtitle duration in clips with a known language soundtrack (English).

We also found a main effect of language in all eye tracking measures except for revisits (see Table 19). Spanish people made significantly more fixations on subtitles than English people, *p =* .001, 95% CI [.31, 1.46], and had a significantly longer mean fixation duration than Polish people, *p* = .025, 95% CI [2.73, 52.20]. They also dwelled the longest in the subtitle, as shown by their longest absolute reading time compared to the English, *p* = .007, 95% CI [49.88, 389.01] and to the Polish, *p* = .006, 95% CI [57.46, 413.62]. Their proportional reading time was longer than analogous time spent by English, *p* = .002, 95% CI [.03, .17] and Polish participants, *p* = .005, 95% CI [.02, .17], see Fig 6.

**Table 19:** Eye tracking results for between-subjects effects in Experiment 2.

Eye tracking measure	df	*F*	*p*	$\eta_{p}^{2}$
Fixation count	2,61	7.209	.002*	.191
Mean fixation duration	2,61	4.474	.015*	.128
Absolute reading time	2,61	6.981	.002*	.186
Revisits	2,61	.405	.669	.013
Proportional reading time	2,61	7.808	.001*	.204

Polish participants had a shorter mean fixation duration than English, *p* = .045, 95% CI [-49.37, -.4], and Spanish participants, *p* = .025, 95% CI [-52.20, -2.73]. They also spent less time with the subtitles than Spanish, as shown by their absolute reading time, *p* = .006, 95% CI [-413.62, -57.46], and proportional reading time, *p* = .005, 95% CI [-.17, -.02].

#### Reading experience

**Fig 7:**
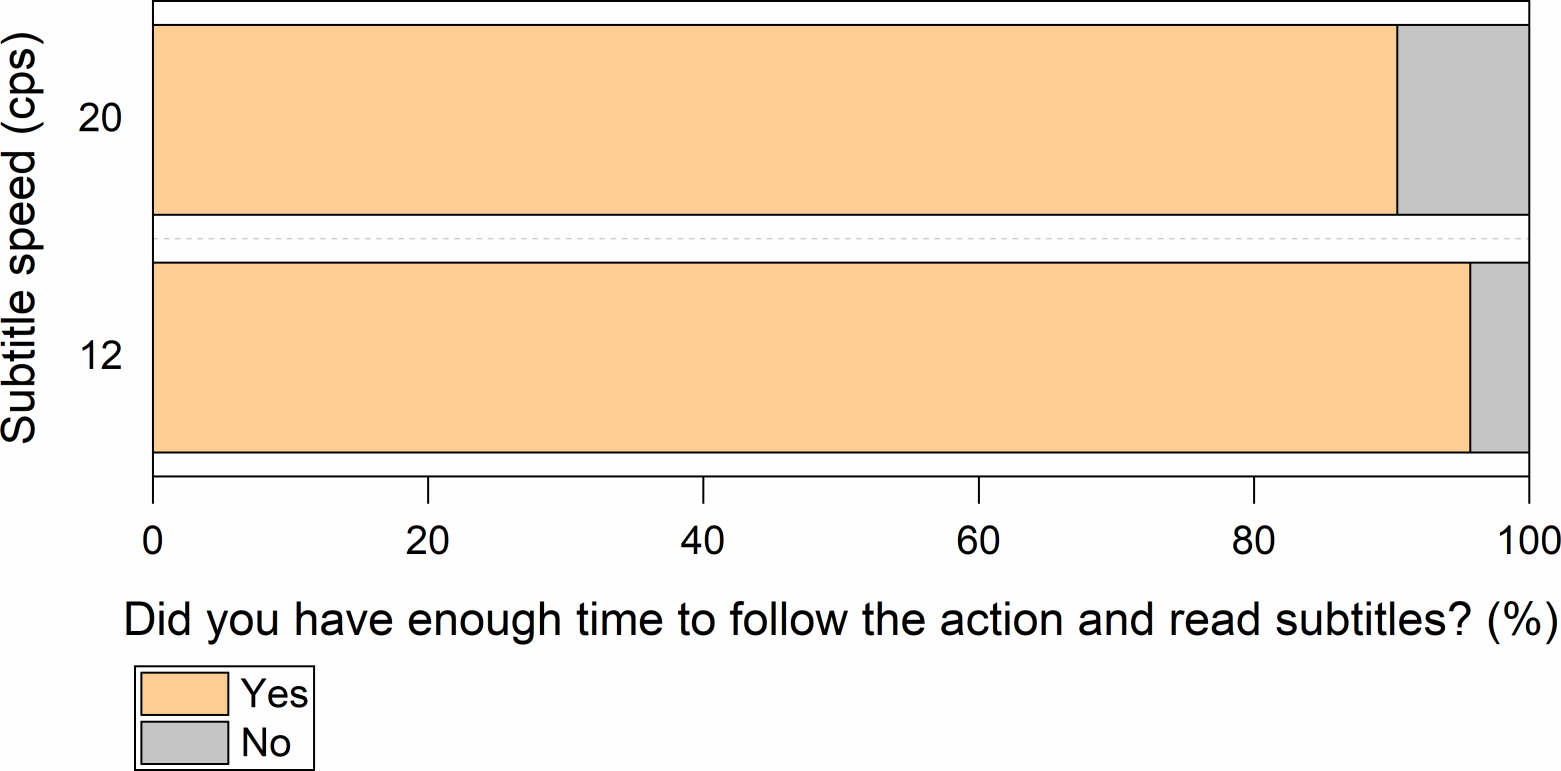
Perceived time to follow the action in clips with a known language soundtrack (English). Participants had to choose one of two options: (1) *I had enough time to read the subtitles and follow the action on screen*, (2) *I didn’t have enough time to follow the action on screen as I was reading the subtitles*.

Most participants declared that in both conditions the subtitles were on the screen for ample time to be read (Fig 6), allowing people to read them and to follow on-screen action (Fig 7).

Interestingly, in contrast to Experiment 1, more people declared to prefer subtitles containing as much dialogue as possible rather than condensed subtitles (see Table 20), although the difference in preference was only about 10%.

**Table 20:** Self-reported reading experience in clips in English.

	12 cps	20 cps
When watching this clip…		
I missed many words	26.6	25.5
I missed some of the words	11.7	16.0
I didn’t miss many words	23.4	24.5
I didn’t miss any words	38.3	34.0
Re-reading		
I re-read many subtitles	1.1	1.1
I re-read some of the subtitles	18.1	7.4
I generally didn’t re-read the subtitles	31.9	40.4
I didn’t re-read any of the subtitles	48.9	51.1
Preferences		
I prefer subtitles to contain less text so that I have more time to read them	44.7	46.8
I prefer subtitles to contain as much dialogue as possible. even if it means they appear and disappear very fast	55.3	53.2

*Note*. The data are given as a percentage of the people who selected a given answer

#### Correlations

Triangulating the self-reports with eye tracking data, we found that in the 12 cps condition, there was a significant relationship between self-reported effort and mean fixation duration, *r*_*s*_(64) = .296, *p* = .002. The effort reported by participants correlated with mismatches they noticed, *r*_*s*_(74) = .296, *p* = .010. There was also a strong correlation between frustration and mismatches, *r*_*s*_(74) = .579, *p* < .000, indicating that the more mismatches noticed, the higher the frustration. We also found a negative relationship between the declared enjoyment and mismatches, *r*_*s*_(74) = -.396, *p* < .000, and with frustration, *r*_*s*_(74) = -.344, *p =* .003. In the 20 cps condition, mismatches were correlated with self-reported difficulty, *r*_*s*_(74) = .352, p = .002, effort, *r*_*s*_(74) = .231, p = .047, and frustration, *r*_*s*_(74) = .422, p < .000. Mismatches were also negatively correlated with enjoyment, *r*_*s*_(74) = -.307, *p* = .008. Overall, this means that the higher the number of mismatches noticed, the lower the enjoyment and the higher the cognitive load reported by the participants.

#### Discussion

Similarly to Experiment 1 and contrary to our predictions, speed had no effect on comprehension. It also did not affect effort, difficulty, or scene recognition. Participants declared to have had enough time to read the subtitles and to follow the on-screen action in both conditions. In contrast to Experiment 1, however, more people wanted to have a full version of the dialogue in the subtitles rather than reduced one.

Slower subtitles displayed at 12 cps were found to be more frustrating and less enjoyable. Participants noticed more mismatches between the dialogue and the subtitle text in the 12 cps condition. Slow subtitles also induced more fixations and higher mean fixation duration, implying more processing effort than faster subtitles.

#### Interviews

Many participants stated that rather than speed, the most important aspect of subtitle quality for them was synchronization: subtitles should appear on the screen synchronously with the onset of speech and disappear when the characters stop talking. In slow subtitle speeds, synchronization is often compromised to allow viewers sufficient time to read the subtitle, which means that subtitles sometimes precede the onset of speech and remain on the screen slightly longer than the actual utterance. In consequence, although the participants did not relate their remarks directly to subtitle speed, they expressed most reservations on slow subtitles, which were perceived as badly synchronized with the dialogue, and in Experiment 2 as not fully reflecting what was said. This also shows a general lack of awareness of professional subtitling rules, where text condensation plays a key role [2, 3], and a popular–yet misguided–perception of subtitles as containing a verbatim version of the dialogues. In fact, a few people voiced their annoyance at text reduction in slow subtitles, saying: “when I hear English, then I actually do compare and I’m getting annoyed when I see too much editing because, to me, it’s cheating people out of the information given, you know, what actually is being said” and “they annoyed me because the subtitles didn’t match with the text, so it felt there was a conflict when I was reading the subtitles and it was just that it annoyed me so much that I stopped”. This, of course, was the case only in Exp. 2 where the clips were in English. Some people suggested that condensation of text in subtitles should be dependent on genre, with documentaries requiring less condensation and more detailed information than feature films.

With regard to the differences between clips with a soundtrack in a familiar and unfamiliar language, the following quote from an English participant best captures the views expressed by many people from all language groups: “When I’m watching something I understand I’d like to be able to read exactly what I’m hearing, and when I’m watching something that I don’t understand I prefer there to be minimal words in the subtitles so that I can get a gist of what’s being said and still be able to follow the scenes of the movie.”

## General discussion

By conducting the two experiments reported in this paper, we examined the following questions: (1) Can viewers keep up with fast subtitle speeds, (2) What is the impact of the language of the soundtrack on subtitle processing, and (3) How does experience with subtitling affect the way viewers from different countries watch subtitled videos? Several novel effects were observed.

### Impact of subtitle speeds

One of the most important findings of this study is that viewers were able to cope even with very fast subtitle speeds: they had sufficient time to read the subtitles and to watch the on-screen action, as evidenced by their comprehension scores, scene recognition results and self-reports on reading experience. As these results were found in all groups of participants in the current study regardless of their native language, we believe that this may be taken to confirm the subtitle effectiveness hypothesis [63–65], according to which subtitle viewing is cognitively effective and there is no “tradeoff between image processing and subtitle processing” [64].

The fact that slower subtitles did not result in higher comprehension may be somewhat surprising but possibly suggests that viewers can cope well with reading subtitles irrespective of their speed. Our results are consistent with the prior work on SDH, which showed that slow edited subtitles did not result in higher comprehension than fast unreduced subtitles [6, 36].

Tracing people’s eye movements allowed us to discover that slow subtitles induced higher mean fixation duration, which is an indication of higher cognitive load [66]. This result may stem from at least two factors. Firstly, it is plausible that slow subtitles–owing to the higher extent of text reduction–were less internally cohesive than fast subtitles and, as such, more difficult to process. This finding is supported by [38], reporting that “subtitles containing more cohesive devices may be easier to process because of their linguistic coherence as well as their cohesiveness with the film text”, as well as by a study on SDH, which found that text editing was associated with less explicit coherence relations and changes in implied meaning [37]. Secondly, longer mean fixation duration in slow subtitles may also be attributed to the discrepancies between the text in the spoken dialogues and the text in the subtitles, particularly in Experiment 2 with English clips. This is because the participants were comparing the subtitles with the dialogues, trying to map the sounds onto the words and to integrate the auditory information with the written text. Upon encountering a mismatch between the two, their cognitive effort increased. As there were more mismatches in slow subtitles compared to fast ones, this negatively affected the mean fixation duration in the 12-cps condition. Mismatches also negatively affected enjoyment and increased frustration levels reported by participants.

The analysis of subtitle reading times with eye tracking has revealed that faster subtitles were read more efficiently than slow subtitles. The reason for this is that even though slow subtitles were displayed on the screen for a longer time than fast subtitles, people did not seem to benefit from the extended display times. For instance, slow subtitles (12 cps) from *Mad Men* were displayed on screen for the total time of 270,520 ms, whereas the medium-paced 16 cps subtitles for 252,800 ms and the fastest 20 cps subtitles for 216,480 ms (when we add up the durations of all the subtitles in the clip). Taking the total clip duration (308,000 ms) as 100%, 12 cps subtitles were on the screen for 88% of clip duration whereas 16 cps subtitles for 82% and 20 cps for 70% of the clip. In other words, slow subtitles stayed on the screen for a longer time than fast subtitles even though they contained less text. At the same time, proportional reading time, i.e. the percentage of time people spent on reading the subtitles as a function of the subtitle display time (taking as 100% the total duration of the subtitles, not the clip), has shown that people spent about 50% of the subtitle display time reading slow subtitles, about 55% reading medium-paced subtitles and about 60% on reading fast subtitles in Experiment 1, and about 40% reading slow subtitles and about 45% reading fast subtitles in Experiment 2. This means that as slow subtitles were displayed longer, people did spent more time reading them in absolute terms, but they still did not look at the subtitles for about 50% of the time the subtitles were on screen. We think the reason why people did not look at the subtitles for about half of the time is that they did not need them to be displayed for that long, as they managed to read them faster. Previous research has shown that subtitles are great gaze attractors [6] and that reading subtitles is automatic [52], so when subtitles are displayed on screen, people automatically look at them (and slow subtitles were there for a long time, attracting people’s gaze for longer). The fact that when watching clips with fast subtitles, people generally spent about half of their time on the subtitles means that they could use the remaining half to follow on-screen action. This seemed to be sufficient for them, as declared in the reading experience questions as well as shown in the subtitle and screen recognition questions. Finally, eye movement analyses also enabled us to discover that slow subtitles triggered more revisits to the subtitle area, which we believe can be attributed to the fact that many slow subtitles were “hanging” on the screen for too long. Viewers re-read slow subtitles much more often than the fast ones–a result also reported by a study on SDH [36]. The number of revisits observed in the slow subtitles was also negatively correlated with lower enjoyment and higher difficulty declared by the participants. Our interpretation of this findings is that when viewers make more revisits, i.e. when they keep moving their eyes down to the subtitle area only to find the same subtitle there, the subtitle reading process may be disrupted and viewers’ suspension of disbelief may be negatively affected.

The results of the current study did not confirm the predictions that fast subtitles would make people spend too much time reading the subtitles to the detriment of the images. This issue ties up with the concept of viewing speed [67] with regard to SDH. Viewing speed is directly determined by the subtitle speed: the faster the speed of subtitles, the more time is spent on reading them. According to Romero-Fresco [67], a subtitle speed of 120 wpm (10–11 cps) will result in approximately 40% spent on subtitles and 60% on images, whereas with fast speeds like 200 wpm (17–18 cps) viewers will spend approximately 80% time on subtitles and only 20% time on images. These predictions were not confirmed in the current study. In Experiment 1, people spent about half of their time reading subtitles and the other half following the filmic image in the 12-cps condition, and about two-thirds of the subtitle display time reading the subtitles in the 20-cps condition. In Experiment 2, possibly because they could understand the language of the soundtrack, viewers spent less than half of their time reading the slow subtitles, and about half of the time reading the fast subtitles. Our results converge with those reported in [43], where the proportion of time spent in the subtitle area during the subtitle display time varied between 32%-66% (*M* = 44%). All in all, these findings suggest that fast subtitle speeds do not necessarily hold viewers back from watching the filmic image.

Similarly to Koolstra et al. [15] and d'Ydewalle et al. [16], we found that absolute reading time was longer in the case of slow subtitles and that proportional reading time increased together with a rise in subtitle speed. One possible interpretation of this is that viewers adjust their reading to the speed of the subtitles: the slower the subtitles, the slower the reading, and vice versa: the faster the subtitles are displayed, the faster the viewers will read them, provided that the speed remains constant throughout the clip: “If the ratio between the amount of text and the time of exposure remains constant, the resulting reading speed will also remain constant” [68].

Subtitle speed did not have any effect on enjoyment in clips in a language unknown to participants. However, when watching English clips, participants’ enjoyment was generally higher in clips with faster unreduced subtitles. Although we asked the participants specifically to focus on the subtitles, it is possible that their enjoyment was influenced by “affect-driven affiliations with media characters” [69], i.e. the way they felt about the characters and the plot as a whole. Given that the notion of enjoyment is very subjective, the differences we observed may be attributed to film genre rather than speed, which was indeed the case in Exp. 1. We acknowledge that it may be difficult to disentangle the effects of enjoying particular films from enjoying clips with a particular subtitle speed.

Post-test interviews with participants allowed us to discover people’s opinions and attitudes towards subtitle speed. Contrary to our expectations, most people were not bothered by the speed of subtitles but instead cared more about subtitle synchronization and subtitle content reflecting the dialogue.

Taken together, our findings on subtitle speeds suggest that the long display time of slow subtitles in this study, equivalent to the six-seconds rule, is unnecessarily long for modern viewers. This carries direct implications on current subtitle practices and subtitle training in the future.

### Impact of the soundtrack

To explore the differences in subtitle processing depending on the film soundtrack, we showed people clips in a language they did not know (Exp. 1), and in a language they were proficient in (Exp. 2). Admittedly, subtitles are traditionally geared towards people who do not understand the language of the soundtrack, but the reality of the audiovisual translation market is that a vast majority of theatrical productions are released in English [50], which makes Exp. 2 particularly relevant and ecologically valid. Important differences that we observed between the two experiments bring us closer to detangling the relationship between film soundtrack and subtitle reading. First, after watching clips in an unknown language, our participants achieved lower results in the comprehension test (on average 80%) compared to clips with the soundtrack in a language they could understand (88%). This shows that viewers integrate information from multiple sources to make sense of subtitled videos and points to the importance of sound in processing. Second, when watching videos in Hungarian, participants declared a slightly higher cognitive load (difficulty and effort) than in the case of videos in English, which means their cognitive processing was more effortful, as they were trying to make sense of the film without the support from the soundtrack. Finally, the fact that people spent more time reading subtitles in the case of clips in Hungarian than in English indicates that viewers support their viewing by making sense of the comprehensible auditory information from the dialogue. On the one hand, given an increasingly high proficiency in English among audiences around the globe, one may argue that “partial knowledge of English mitigates losses resulting from subtitle condensation” [50]. On the other hand, as viewers are mapping the sounds from the dialogues onto the words in subtitles when they know the language of the soundtrack, any discrepancies between the two may disturb their viewing process. This was also demonstrated in a study where viewers made more revisits to the subtitle in the clip using non-literal translation of the dialogue [70].

The implication our findings may have on current subtitling practices is that with films whose language is well understood in the target country, subtitles could contain more text and be displayed at faster speeds, whereas in the case of films with lesser known languages, more text reduction and slower speeds may be necessary.

### Impact of experience with subtitling

Our findings demonstrate the previous experience with subtitling may affect the way people process subtitled videos. For example, Spanish participants, who had been most exposed to dubbing, dwelled longest in the subtitle area in both experiments and had longest mean fixation duration–a measure usually attributed to “more effortful cognitive processing” [66]. Together with longer absolute reading time and higher fixation count, it is plausible that Spanish people had to expend more effort into reading subtitles than the two other groups. On the other hand, Polish people spent the least amount of time reading the subtitles, indicating they read them faster. English participants declared to experience a much higher cognitive load compared to Polish and Spanish people, which we believe can be attributed to their smallest previous exposure to subtitling. Yet, despite the above differences, all groups seem to have processed subtitles fairly efficiently, showing that subtitles can be used “even with populations who are less familiar with it” [63].

## Conclusion

The current article examined the effects of subtitle speed on viewers’ processing of subtitled videos. The most important result was that people could cope well with fast subtitle speeds and that they preferred them to slow ones in English clips. These results provide empirical grounds for revisiting current subtitling guidelines and audiovisual translation industry practices.

Further research is required to fully understand the nature of the impact of the soundtrack and of previous experience with subtitling on subtitle processing observed in the current study. Other issues that need to be addressed include disentangling the impact of subtitle speed from that of text editing, investigating the role of language proficiency [71], film genre and film complexity [72] in subtitle processing, examining audience preferences on the faithfulness of subtitle translation [70] as well as replicating our results on speakers of other languages and age groups. Examination of these issues will allow a greater understanding of the subtitle processing and the development of empirically grounded subtitling rules, meeting the needs of a dynamically growing number of subtitle end users.
